# Neural correlates reveal sub-lexical orthography and phonology during reading aloud: a review

**DOI:** 10.3389/fpsyg.2014.00884

**Published:** 2014-08-12

**Authors:** Kalinka Timmer, Niels O. Schiller

**Affiliations:** ^1^Department of Psychology, York UniversityToronto, ON, Canada; ^2^Leiden University Centre for Linguistics, Leiden UniversityLeiden, Netherlands; ^3^Leiden Institute for Brain and Cognition, Leiden UniversityLeiden, Netherlands

**Keywords:** reading aloud, masked priming paradigm, grapheme-phoneme conversion, reading modeling, ERPs (event-related potentials)

## Abstract

The sub-lexical conversion of graphemes-to-phonemes (GPC) during reading has been investigated extensively with behavioral measures, as well as event-related potentials (ERPs). Most research utilizes silent reading (e.g., lexical decision task) for which phonological activation is not a necessity. However, recent research employed reading aloud to capture sub-lexical GPC. The masked priming paradigm avoids strategic processing and is therefore well suitable for capturing sub-lexical processing instead of lexical effects. By employing ERPs, the on-line time course of sub-lexical GPC can be observed before the overt response. ERPs have revealed that besides phonological activation, as revealed by behavioral studies, there is also early orthographic activation. This review describes studies in one’s native language, in one’s second language, and in a cross-language situation. We discuss the implications the ERP results have on different (computational) models. First, the ERP results show that computational models should assume an early locus of the GPC. Second, cross-language studies reveal that the phonological representations from both languages of a bilingual become activated automatically and the phonology belonging to the context is selected rapidly. Therefore, it is important to extend the scope of computational models of reading (aloud) to multiple lexicons.

Reading aloud is executed without much conscious thought, though it requires complex underlying processing for correct execution. The process can be divided into three general steps. The first step is visual word recognition. This step constitutes the identification of letter features followed by letter and grapheme identification, which finally results in the identification of the whole word (e.g., Ferrand and Grainger, 1992; Grainger and Ferrand, 1994; Frost, 1998; Carreiras et al., 2005; Grainger, 2008). The second step is the conversion of the orthographic representation into a phonological representation (i.e., grapheme-to-phoneme-conversion; GPC). The final step concerns the actual overt production of the printed word, the conversion of the orthographic or phonological representation into a phonetic code that activates the corresponding articulatory-motor program (Browman and Goldstein, 1988).

The goals of this review are: (1) to give insight into the time course of sub-lexical activation of orthography and phonology during reading aloud, (2) identify the locus of GPC, and (3) to propose how ERP results can inform computational models. This topic has mostly been investigated with the lexical decision task (LDT) combined with the masked priming paradigm (e.g., Carreiras et al., 2005; Grainger and Holcomb, 2009). In masked priming, a visible target stimulus is preceded by the brief presentation of a prime stimulus to avoid strategic processing (Forster, 1998). It is assumed that the sub-lexical segments of the masked prime are activated. When the target is presented and segments match, they are pre-activated and brain activity reaches a specified threshold faster (Horemans and Schiller, 2004). However, LDT (i.e., deciding whether stimuli are words or non-words) is strongly influenced by lexical factors such as word frequency, familiarity, and neighborhood size (Balota et al., 2004). Therefore, it cannot be ascertained that *sub-lexical* GPC is captured. When reading aloud is combined with masked priming the lexical effects are minimized. This mini-review focuses mainly on the latter research to capture sub-lexical GPC. It also focuses on electrophysiological measures to demonstrate the online time course of the GPC process before the endpoint of processing (captured by behavioral data).

Below, the computational models that simulate reading (aloud) will be described. Next, behavioral and event-related potentials (ERPs) studies revealing the underlying processes of reading aloud are discussed. Lastly, we discuss how well the models account for the behavioral and ERP findings in the literature.

## MODELING THE READING PROCESS

**FIGURE 1:**
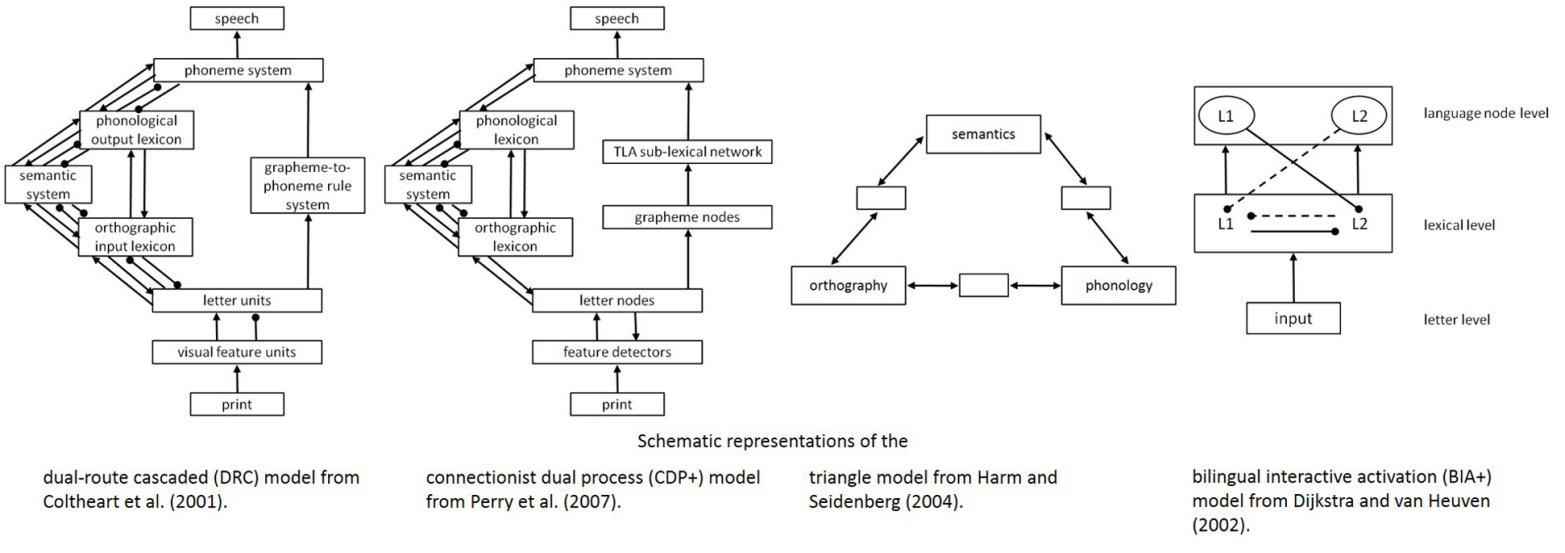
**Schematic representations of computational models of reading (aloud) and speech production**.

Models of reading can be distinguished based on the locus of the GPC process: early or late. However, the early models modulate GPC rules in slightly different manners. For instance, according to the *dual-route cascaded* (DRC) model, in the lexical route, the phonology of a written word is retrieved as a whole (*parallel*) from the mental lexicon. In the non-lexical route, graphemes are transferred one by one (*serial*) into corresponding sound codes (i.e., phonemes) on the basis of GPC rules. From beginning-to-end of a word^1^, a rule is found for translating each letter into a phoneme (e.g., <c> is pronounced as /s/ when the following letter is a front vowel like <e, i, y>, and pronounced as /k/ when the following letter is a back vowel, like <o, a>). Non-word reading can only be simulated by the non-lexical route because non-words do not have a lexical entry. Irregular words (e.g., *pint*) can only generate a correct pronunciation in the lexical route (i.e., correct /paınt) as the non-lexical route follows standard GPC rules (i.e., incorrect /pínt/). This causes competition between the routes, which slows down processing for irregular compared to regular words that have matching pronunciations for both routes (see **Figure 1**; Coltheart et al., 2001; Mousikou et al., 2010). Evidence for serial processes comes from the beginning-to-end nature of the regularity effect, which demonstrates longer naming latencies for irregular words that have exceptional spelling early in the word (e.g., *pint*) compared to late in the word (e.g., *debris*; Coltheart and Rastle, 1994; Rastle and Coltheart, 1999). Additional evidence comes from position dependency of the masked onset priming effect (MOPE) for onset-related but not offset-related prime-target pairs (Forster and Davis, 1991; Kinoshita, 2000; Schiller, 2004).

Another dual-route computational model, the *connectionist dual process* [CDP(+)+] model, has taken the lexical route from the DRC model. However, the non-lexical route is not rule based like the DRC model, but instead a simple two-layer network is employed. In this network the input layer represents the written word and the output layer represents the phonological representation belonging to the written word. The network is trained on grapheme-phoneme correspondences, through a graphemic buffer, which means not only single letters but also multi-letter graphemes are represented (e.g., longer graphemes are preferred over shorter: <kn> over <k> to read knife; context sensitivity for <c> in carpet; Perry et al., 2007, 2010). The correct pronunciation is chosen in the phonological output buffer where the pronunciations of both routes come together (i.e., lexical route /paınt/; non-lexical route /pınt/; see **Figure 1**).

The triangle model suggests orthography can be linked to phonology directly or mediated by semantics. Similar to the CDP++ model, in the triangle model orthographic input units are converted to phonological output units through hidden units during a training period. After the conversion of each word, the phonological output is compared to the phonological target word. Based on error for the output units, weights are updated to reduce the error. Thus, irregular GPC correspondences are learned through consistency (Harm and Seidenberg, 2004; see **Figure 1**).

The MOPE is believed to reflect the GPC process. Models assuming an early locus of GPC suggest that a MOPE will only be present for low-frequency words and the effect will disappear when applying a lexical decision or conditional naming task (i.e., only naming real words while they are intermixed with non-words; Forster and Davis, 1991; Coltheart et al., 2001; Kinoshita and Woollams, 2002). However, this idea is not unchallenged; the speech-planning account has suggested a later locus of GPC, namely during the segment-to-frame association part of speech-planning (Kinoshita, 2000; Kinoshita and Woollams, 2002). This process involves the retrieval of a word’s phonological segments and combining them with the metrical frame of a word (e.g., number of syllables and stress pattern) to create the speech plan necessary for speech production (Levelt et al., 1999). The MOPE is explained by a conflict from mismatching onset phonemes between prime and target holding up the segment-to-frame association process. Other behavioral data, like the regularity effect, can also be explained by a hold up in the segment-to-frame association process (Kinoshita, 2000; Kinoshita and Woollams, 2002).

The models described above only address reading in one language. However, next to within-language priming, cross-language phonological priming reflects fast and automatic activation of both the first (L1) and second language (L2) GPC rules (Dijkstra and van Heuven, 2002). The *bilingual interactive activation* (BIA) model (Dijkstra and van Heuven, 2002) can explain L2–L1 cross-language priming effects because it assumes a single lexicon in which words from the different languages a bilingual speaks are simultaneously activated and interconnected (i.e., language non-specific selection) but can also mutually inhibit each other. This is necessary because when bilinguals speak in one language, they must inhibit words from the non-target language to avoid interference. Current research supports the notion that at the lexical level, our languages are represented together (i.e., non-selectively), which is supported by research showing cross-language competition and language switching costs (see **Figure 1**; Dijkstra and van Heuven, 2002). However, is it only the lexical level where all languages are represented collectively or does it extend to the sub-lexical orthographic and phonological level?

## BEHAVIORAL FINDINGS

Models of reading suggest different loci of GPC: sub-lexical or during speech preparation. Facilitation for shared onset segments (i.e., called MOPE in priming paradigms) during reading aloud tasks (i.e., word naming; e.g., Forster and Davis, 1991; Kinoshita, 2000, 2003; Kinoshita and Woollams, 2002; Schiller, 2004, 2007, 2008; Malouf and Kinoshita, 2007), implicit priming studies (e.g., Meyer, 1991; Damian and Bowers, 2003; Alario et al., 2007), picture–word interference (PWI) tasks (e.g., Schriefers et al., 1990; Meyer and Schriefers, 1991), and color–object picture naming tasks (Damian and Dumay, 2007, 2009), but not during LDT (Forster and Davis, 1991; Grainger and Ferrand, 1996; Carreiras et al., 2005) or conditional naming (Kinoshita and Woollams, 2002) could suggest that GPC facilitation effect occurs during speech preparation as this step is not necessary for non-production tasks (speech-planning account; Kinoshita, 2000; Kinoshita and Woollams, 2002). However, models assuming an early locus of GPC can explain the absence of a MOPE during LDT by means of an overall slowdown during the lexical decision process which dissolves any earlier facilitation effects.

In a similar manner, opaque Persian words (i.e., words containing short vowels *not* marked in the spelling; e.g., 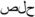 */solh/*; *peace*), just as English irregular words (e.g., *pint*), require lexical knowledge to be read aloud correctly (Baluch and Besner, 1991) and do not show a MOPE (Timmer et al., 2012). The conflict between the incorrect pronunciation (i.e., /pínt/) in the non-lexical route and the correct pronunciation (i.e., /paınt/) in the lexical route slows down processing and dissolves earlier facilitation effects, like the MOPE, according to the DRC model (Coltheart et al., 2001; Mousikou et al., 2010). For transparent Persian words (i.e., words containing long vowels which are marked; e.g., 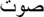 /sot/; *voice*) there is no competition between routes, and no slowdown occurs, therefore revealing a MOPE just like regular English words do. The speech-planning account explains these effects by a holdup during the segment-to-frame-association of speech-planning. For example, conditional naming (i.e., only naming words but not non-words) latencies are slower than when all words are named. The time criterion is set at a point in time that is appropriate for the type of words to be named correctly. A later time criterion could also be adopted for irregular words. The later time criterion for irregular words could dissolve the effect of matching onset segments (Kinoshita, 2000; Kinoshita and Woollams, 2002). Thus, these behavioral results cannot differentiate between the different accounts on the locus of the MOPE. One way to investigate whether GPC has an early (DRC and CDP++) or late (speech-planning account) locus is by employing an electrophysiological measure that can determine the time-course on the millisecond from target presentation to overt production.

Furthermore, behavioral results suggest that the MOPE, reflecting GPC, is phonological in nature; words that match on orthography (but not phonology; e.g., *circle* – *CARPET*), show similar response latencies compared to an unrelated prime-target pair (e.g., *powder* – *CARPET*). However, response latencies are faster when phonology, rather than orthography, is matched (e.g., *kernel* – *CARPET*) compared to the unrelated condition (e.g., Schiller, 2007; Mousikou et al., 2010; Timmer and Schiller, 2012; Timmer et al., 2012). The above literature demonstrating segmental priming (MOPE) employed alphabetic languages. Syllabic languages (e.g., Mandarin and Chinese), however, only show facilitation with full syllable overlap during reading aloud (Verdonschot et al., 2011), implicit priming (Chen et al., 2002), PWI (Wong and Chen, 2008, 2009), and color–object naming (Qu et al., 2012). This difference may be due to the writing system (i.e., each character represents a syllable) or syllable structure (i.e., simpler in syllabic languages; Davenport et al., 2010).

Above, we have interpreted the findings in light of L1 research. However, the phonological MOPE presents not only in one’s L1 but also in one’s L2 (Timmer and Schiller, 2012), and even in cross-language contexts (Jouravlev et al., 2014; Timmer et al., 2014a,b). For example, an L2 (English) prime that was phonologically related to the onset of an L1 (Dutch) target (e.g., *phone* – *FIETS*) revealed faster response latencies compared to an unrelated condition (e.g., *pain* – *FIETS*). These results suggest that both L1 and L2 phonology become rapidly activated from a masked prime while performing a task in the L1. In addition, under certain circumstances Mandarin–English bilinguals reveal segmental, instead of only syllabic, priming during Mandarin reading due to their knowledge of an alphabetic language (Verdonschot et al., 2013).

## ELECTROPHYSIOLOGICAL TIME COURSE

The measure of ERPs provides empirical answers to the locus of the MOPE by providing an on-line time course of the reading process. The visual word recognition literature (LDT) has associated the N250 ERP component with sub-lexical GPC (e.g., Grainger et al., 2006; Holcomb and Grainger, 2006; Carreiras et al., 2009; Grainger and Holcomb, 2009; Midgley et al., 2009; for an overview see Grainger and Holcomb, 2009). However, that LDT promotes lexical processing. In addition, large prime-target overlap has often been used (e.g., *conal* – *CANAL* or* brane* –* BRAIN*) which also promotes top-down processing and cannot suggest sub-lexical processing. Therefore, studies using reading aloud and only segmental onset overlap provide stronger evidence for the time course of sub-lexical GPC, presenting itself as a negative ERP component between 80 ms and up to 200 ms after target presentation (Timmer and Schiller, 2012; Timmer et al., 2012). The peak occurred around 150 ms during reading instead of 250 ms during LDT. The reading aloud literature is in line, though slightly earlier, with a meta-analysis of word naming, proposing that the GPC process occurs approximately 150–330 ms after target presentation and not a late locus of GPC during speech preparation, as proposed by the speech-planning account (Kinoshita, 2000; Kinoshita and Woollams, 2002), within the 330–600 ms time window (Indefrey and Levelt, 2004; Indefrey, 2011).

**FIGURE 2:**
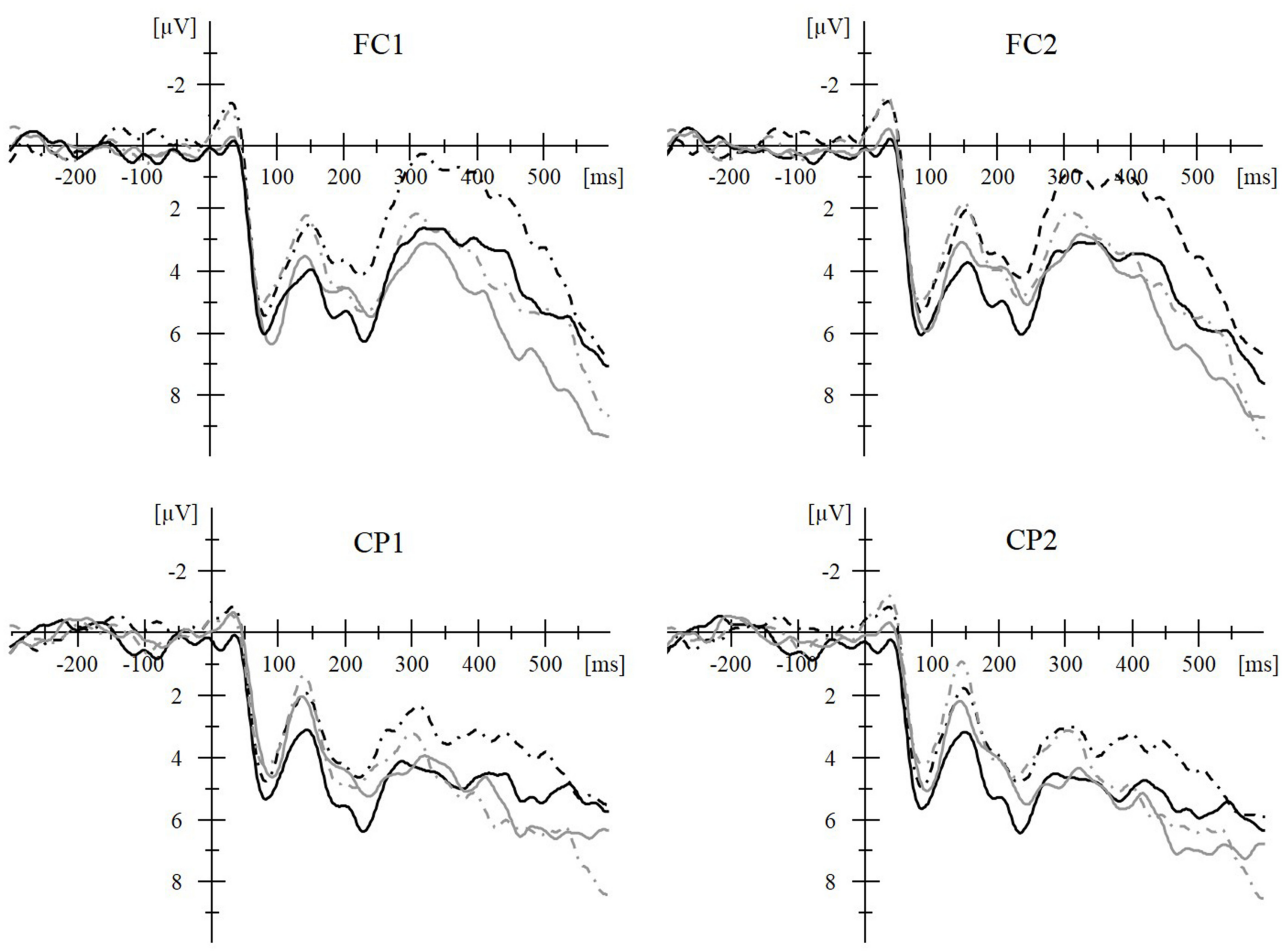
**Averaged stimulus-locked event-related potential (ERP) waveforms during a masked priming paradigm demonstrate the online time course of grapheme-to-phoneme-conversion (GPC) during reading aloud.** Transparent words (i.e., words with marked vowels) are represented by black lines and opaque words (i.e., words with vowels not marked) by gray lines. For both word types, more negative amplitudes for phoneme-mismatch (O-P-; e.g., respectively, 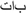 /ta:b/ “swing” – 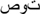 /sot/ “voice” and 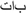 /ta:b/ “swing” – 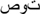 /sot/ “voice”; dashed lines) than phoneme-match (O-P+; e.g., 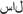 /sa:l/ “year” – 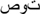 /sot/ “voice” and /sa:l/ “year” – 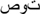 /sot/ “voice”; solid lines) conditions in the 80–160 ms time window demonstrate phonological priming. This supports an early locus of GPC for both word types. During the 300–480 ms time window, this effect is only continued for the transparent words. This later time window might reflect processing in the output buffer. The multiple pronunciation options for Persian opaque words slow down processing and eliminate the masked onset priming effect (MOPE) for opaque words in both the ERPs and the behavioral results. To conclude, ERPs demonstrate that in spite of the discrepancy in behavioral measures all word types have an early GPC. (A 20 Hz filter was applied for the clarity of the waveforms.) This Figure has been published before in Timmer et al. (2012).

Even stronger support for the early locus of GPC comes from the presence of phonological priming for *both* transparent and opaque Persian words in the 80–160 ms time window, though only transparent words showed a MOPE behaviorally. During the 300–480 ms time window, phonological activation is only found for transparent Persian words (Timmer et al., 2012; **Figure 2**). This supports the DRC, CDP ++, and triangle models where GPC takes place early during the non-lexical route. When the two routes come together, in the output buffer, the multiple pronunciation options for Persian opaque words slow down processing and eliminate the MOPE for opaque words in both the ERPs and the behavioral results. Just like opaque words (Timmer et al., 2012), irregular words (Kinoshita and Woollams, 2002) and unpronounceable strings of consonant (Dimitropoulou et al., 2010) do not show a MOPE behaviorally. Based on the behavioral absence of a MOPE for opaque words but its presence in the early ERPs we would also expect early ERP effects for irregular words and unpronounceable non-words in possible future endeavors.

Further, behavioral data demonstrated phonological, but not orthographic facilitation, suggesting that the MOPE is phonological in nature. However, ERPs revealed both orthographic and phonological activation during the N250 component without an amplitude or latency difference in onset. This suggests automatic and rapid GPC during reading aloud (Timmer and Schiller, 2012; Timmer et al., 2012). Phonological activation has a frontal distribution which is in line with an fMRI meta-analysis showing more left inferior parietal activation for pseudo- than words indicating more effortful processing during the non-lexical route for pseudo-words (Taylor et al., 2013). In contrast, LDT literature usually shows phonological activation (250–350 or 350–450 ms) after orthographic activation (150–250 ms; Grainger et al., 2006; Carreiras et al., 2009). Later phonological activation for LDT could occur because retrieving phonological codes during silent reading is non-essential. Another possibility is that the large prime-target overlap strengthened lexical phonological effects. Interestingly, behavioral research revealed that segment-only overlap was not enough to facilitate priming in syllabic languages; however, ERPs revealed that Mandarin speakers did process the segment initially (Qu et al., 2012). When a picture and word shared all phonological segments except the consonantal onset, no phonological activation presented itself in the ERPs for category associates (Jescheniak et al., 2003) as phonological activation occurs from beginning-to-end of a word.

Cross-language ERP results show orthographic and phonological activation during the same time windows as for L1 research suggesting rapid and automatic activation of the sub-lexical phonology of both the L1 and L2 whereby the phonology belonging to the language of the word is automatically selected (Jouravlev et al., 2014; Timmer et al., 2014a,b). To conclude, these results provide additional evidence for an early locus of the MOPE. However, most computational models cannot account for GPC rules or grapheme-phoneme correspondences from multiple languages within one system.

During reading aloud studies, orthographic and phonological effects continue into later time-windows to different extents. At this moment, it is not entirely clear why the priming effects are maintained at later components in some cases, but not in others. However, LDT research has already shed light on the possible meaning of these later components. The P325 has been associated with lexical form processing; it was demonstrated to be susceptible to partial (e.g., *teble* – *TABLE*) compared to full repetition priming, but not to unrelated compared to partial priming (Holcomb and Grainger, 2006). However, the reading aloud literature has always used unrelated and partial priming and has shown that effects continue into this later component (Timmer and Schiller, 2012; Timmer et al., 2012, 2014a,b; Jouravlev et al., 2014). The N400 is believed to be a form-meaning interface in LDT research (e.g., Holcomb and Grainger, 2006; Grainger and Holcomb, 2009). Future research is necessary to add to the understanding of later components in the reading process.

## DISCUSSION

Behavioral data have not been able to differentiate between models of visual word recognition assuming an early locus of GPC (DRC; CDP+, and triangle model) and a late locus of GPC (speech-planning account). Recent ERP studies have clearly shown GPC occurring approximately 150 ms after target presentation, providing neural evidence for an early locus (Timmer and Schiller, 2012; Timmer et al., 2012, 2014a,b; Jouravlev et al., 2014).

All models assuming an early locus of GPC can explain the within-language phonological MOPE, though each does so slightly differently. For words starting with letters that have multiple print-to-sound associations (e.g., <c> as /s/ or /k/) the DRC model suggests GPC takes place in the non-lexical opposed to the lexical route and is rule-based. For example, context rules assure that the first <c> in *circus* is read as an /s/ because it is followed by a front vowel and as a /k/ in *carpet* because it is followed by a back vowel. In contrast, the CDP+ and triangle model are not rule-based, but train the model on GPC correspondences. The phonological output of the model is compared to the orthographic input and adjusted if necessary, based on, for example, context sensitivity in the CDP+ model. The triangle model adjusts its weights based on erroneous phonological outputs.

The cross-language phonological MOPE currently cannot be accommodated by these computational models as they have not focused on bilingualism. To do so, however, may be possible with some simple modifications. To accommodate for both L1 and L2 GPC, when deviating from each other (e.g., <kn> as /kn/ in Dutch and /n/ in English), this could be handled in a similar manner as is now done for multiple print-to-sound-associations within a language. Instead of using the following vowel as a context, as for only L1 GPC, the language of the word may be used as a context to select L1 or L2 GPC. The DRC model must add additional rules, the CDP++ model must be trained on L2 words, and the triangle model must adjust weights depending on the language of the word. For syllabic languages, Roelofs (2014) adjusted the WEAVER++ model (Levelt et al., 1999) to accommodate the absence of segmental priming. While the lexical word activates all its segments and metrical frame (stress) in alphabetic languages, it immediately activates atonal syllables in syllabic languages. To conclude, the present behavioral and neural results suggest future directions for computational models.

## Conflict of Interest Statement

The authors declare that the research was conducted in the absence of any commercial or financial relationships that could be construed as a potential conflict of interest.

## Footnotes

1 Most alphabetic scripts have a writing system from left-to-right, but most Semitic and Indo-European languages, like Persian, are written from right-to-left.
